# Carbon monoxide levels after inhalation from new generation heated tobacco products

**DOI:** 10.1186/s12931-018-0867-z

**Published:** 2018-08-31

**Authors:** Pasquale Caponnetto, Marilena Maglia, Gaetano Prosperini, Barbara Busà, Riccardo Polosa

**Affiliations:** 1 Centro per la Prevenzione e Cura del Tabagismo (CPCT), Azienda Ospedaliero-Universitaria “Policlinico-V. Emanuele”, Università di Catania, Catania, Italy; 2 Dipartimento di Medicina Clinica e Sperimentale, Università di Catania, Catania, Italy; 3 MCAU Ospedale Cannizzaro, Catania, Italy; 4 UOC Farmacia Ospedaliera, ARNAS Garibaldi, Catania, Italy; 5 Center of Excellence for the Acceleration of Harm Reduction (CoEHAR), Università di Catania, Catania, Italy; 6 UOC di Medicina Interna e d’Urgenza, Edificio 4, Piano 3, AOU “Policlinico-V. Emanuele”, Via S. Sofia 78, 95123 Catania, Italy

**Keywords:** Heated tobacco products, Tobacco-heating products, Combustion, Exhaled breath carbon monoxide

## Abstract

Heated tobacco products (HTPs) are new tech devices that release nicotine and other volatile compounds into an inhalable aerosol by heating the tobacco. At their operating temperatures, tobacco combustion is unlikely.

The aim of this randomized cross-over study was to measure the exposure levels of the combustion marker, carbon monoxide in the exhaled breath (eCO) of subjects after use of two HTPs and to compare these levels with participants’ own brand of cigarettes.

A total of 12 healthy smokers who reported smoking ≥10 conventional cigarettes per day for at least 5 years took part in the study. Product administration consisted of a first round of 10 puffs, which was followed by an identical second round after a 5 min pause in between rounds. After obtaining a baseline eCO value, this measure was recorded at 5, 10, 15, 30, and 45 min after the first puff of the first round. In contrast to combustible cigarettes, no eCO elevations were observed in the exhaled breath after use of the HTPs under investigation in any of the study participants.

## Introduction

The health risks associated with cigarette smoking are well established [1]. The harmful effects of smoking result from chronic exposure to thousands of toxic chemicals and carcinogens in cigarette smoke following the combustion of tobacco in the cigarette.

In conventional cigarettes, tobacco combustion is known to release nicotine together with a multitude of harmful and potentially harmful chemical constituents (HPHCs) including carbon monoxide (CO) [2]. Conversely, products that do not require combustion to deliver nicotine (e.g. nicotine replacement therapies - NRTs, smokeless tobacco, electronic cigarettes) are less likely to release much HPHCs, including CO. For this reason, non-combustible nicotine sources have been proposed for smoking harm reduction [3].

Heated tobacco products (HTPs) - the newest addition to the smoking harm reduction approaches - are an emerging class of nicotine delivery devices that do not burn tobacco. HTPs generally consist of a holder (a battery and an electronically controlled heating element) and a rod (containing processed tobacco, glycerine, and other additives). Newer generation HTPs release nicotine and other volatile compounds by heating the tobacco rod at temperatures not exceeding 350 °C. At these temperatures, tobacco combustion is unlikely and as a consequence far fewer chemical toxicants (including CO) are formed [4]. However, earlier generation HTPs that were marketed as less harmful alternatives to combustible tobacco still produced high levels of carcinogens and more CO than conventional cigarettes [5]. This is in contrast to electronic cigarettes (ECs), which heat a tobacco-free nicotine solution and do not elevate CO levels [6–8].

Studies of subjects who have switched from smoking to the newest generation of HTPs show substantial exposure reductions to a wide range of smoke chemicals [9–12]. However, surprisingly, in two of these studies, when exposure to CO was evaluated by measurement in exhaled breath, a significant elevation was detected [13, 14]. It is possible that combustion can still take place in some HTPs designs.

The aim of this randomized cross-over study was to measure the exposure levels to the combustion marker, carbon monoxide in the exhaled breath (eCO) of subjects after use of two HTPs and to compare these levels with participants’ own brand cigarettes. Smokers will be able to make better choices if they are guided by findings of independent studies focused on the relative reduction in exposure risk after switching to HTPs.

## Methods

A total of 12 healthy smokers (6 Males, 6 Females; mean age of 28.6 yrs), smoking ≥10 conventional cigarettes per day (cig/day) for at least 5 years took part in this randomized cross-over trial. At screening participants provided a baseline eCO ≥ 10 ppm (ppm) and were provided training for at least 30 min. The training focused on use of both HTPs as per manufacturers’ recommendations.

Participants were randomized to use either iQOS (iQos device with Marlboro Heets, Philip Morris International), or GLO (Glo device with Kent Neostik, British American Tobacco), or their own brand conventional cigarettes on three separate study days (separated by at least 48 h). The randomization sequence was computer-generated.

Subjects were asked to refrain from smoking for at least 12 h prior to each study visit. As per eligibility criteria, smoking abstinence was verified upon arrival by eCO measurements ≤ 10 ppm obtained from a single expiratory breath using a hand-held eCO meter (Micro CO; Micro Medical Ltd., UK) according to the manufacturer’s recommendations. Product administration was like that described by Vansickel et al. [6] and consisted of a first round of 10 puffs with a 30-s interpuff interval (puff number and interpuff interval were monitored by study staff) followed by an identical second round after a 5 min pause in between rounds. Fully charged HTPs were used during study sessions. After obtaining a baseline value, eCO was recorded at 5, 10, 15, 30, and 45 min after the first puff of the first round.

## Results

**Fig. 1:**
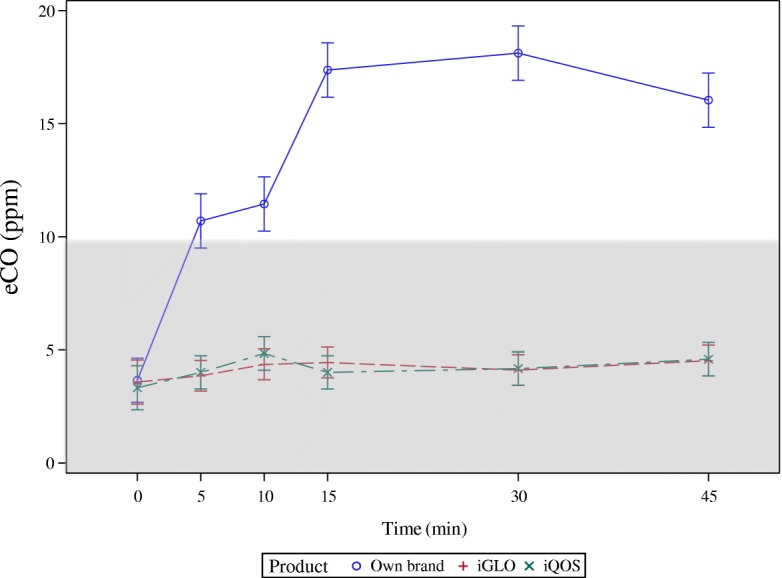
Time trends of eCO levels, separately for each tobacco product use. Time-trends (means ±95% confidence intervals) of exhaled carbon monoxide (eCO) measured at baseline (BL), and at 5, 10, 15, 30 and 45 min after using iQOS (X), GLO (+), and own brand cigarettes (open circle). The model used to produce the repeated measures ANOVA had product and time-point as main effects and it was adjusted by age, sex and baseline measurements. The within-product effect was significant only for own brand cigarette (*p* < 0.0001). The between-product effect was significantly different for both HTPs compared to own brand cigarette (*p* < 0.0001), but no different when iQOS was compared to GLO. Shaded area delineates commonly accepted eCO reference ranges for non smokers

Figure 1 illustrates time trends of eCO levels, separately for each product. Baseline eCO levels were similar among study days and all were below 5 ppm. As expected, large significant within-subject effect (i.e. time, *P* < 0.0001) was found for changes in eCO following participants’ use of their own brand of traditional cigarettes as compared to either HTP. No significant changes in eCO levels were observed after using the two HTPs under investigation; their median eCO level (95%CI) reaching a max peak at 45 min for GLO with 4.5 (3.9;5.2) ppm and at 15 min for iQOS, with 4.9 (4.1;5.6) ppm, respectively. Repeated-measures ANOVA post-hoc comparisons showed significant differences between-product effect (iQOS/GLO vs own brand cigarette; *P* < 0.0001; iQOS vs GLO; *P* = NS) (Table 1).

**Table 1:** Differences of Least Squares Means between Products

Effect	Product	Product	Estimate	Standard Error	DF	t Value	Pr > |t|	Alpha	Lower	Upper
Product	Own brand	GLO	10.4726	0.3126	162	33.51	<.0001	0.05	9.8554	11.0899
Product	Own brand	iQOS	10.4072	0.3198	162	32.54	<.0001	0.05	9.7757	11.0387
Product	GLO	iQOS	−0.06542	0.2270	162	−0.29	0.7736	0.05	−0.5138	0.3829

## Discussion

This is the first independent study investigating eCO levels after use of two recently marketed HTPs. We observed no eCO elevations during inhalational testing with HTPs under investigation in any of the study participants. Our findings concur with findings from e-cigarette studies [6–8] as well as from manufacturer and independent data on HTPs. Findings from tobacco industry reports indicate negligible CO emission (0,436 mg/stick and 0,223 mg/stick for iQOS and GLO, respectively; compared to 30.2 mg/tobacco cigarette, that is 69,3 and 135,4 times less) [13, 14]. Independent analytical chemistry data confirms substantial CO level reductions [15, 16] in HTP aerosol emissions compared to combustible cigarettes. Very large reductions were also reported for other tobacco combustible toxicants including TSNAs, PAHs and aldehydes [15, 16]. In another recent independent report, the yields of the carbonyl compounds formaldehyde, acetaldehyde, acrolein, and crotonaldehyde were 80–96% lower compared to combustible cigarettes [17]. Similarly, the emissions of the volatile and semi-volatile compounds benzene, 1,3-butadiene, isoprene, styrene, and toluene were reduced by 97–99%. It should be noted that smoking machine protocols are standardized methods aimed to assess emissions, but not human exposure. In our study we investigated human CO exposure, by measuring and comparing CO levels in the exhaled breath from smokers puffing their own brand, IQOS and GLO. Dissimilarity in absolute CO values reflects different methodologies used for analysis of emissions and assessment of exposures [18]. Nonetheless, the very large reductions reported in CO emissions [13–17] are fully consistent with our findings in human exhaled breath. Interestingly, in a recent paper about GLO aerosol emission [19] when the tobacco stick was ignited and smoked as a conventional cigarette a sharp increase in CO, NO and CO2 was observed, thereby confirming that it is the burning (and not the heating) of the tobacco that results in combustion. We conclude that there is no combustion in iQOS and GLO. Nonetheless, we cannot exclude the presence of toxins generated through pyrolysis in the aerosol of these HTPs.

When interpreting the study findings, many factors need to be considered. First, findings from a small study should be interpreted with caution. Yet reassuring, a power analysis of the collected data indicated that 12 subjects were sufficient to detect differences of 3 ppm or larger, with a power of 80%. Therefore, our randomized crossover trial was more than adequately powered to detect eCO differences between combustible and non-combustible tobacco products. Moreover, eCO elevations larger than 3 ppm were never reported in any of the 12 subjects during HTP use, and most of the eCO changes measured were within the margin of error of the CO monitoring device. Nonetheless, residual combustion (of no clinical significance) cannot be discounted when human exposure studies with low sensitivity measurements are used. Second, the results of the study are product specific under acute laboratory condition of use and cannot be extended to other HTPs or to the same products under investigation after prolonged use. Third, our results appear to support tobacco industry data and may therefore be considered with reluctance. It is important that data reported by the industry is independently verified, as with this investigation.

In relation to the wider implications of this study, it is our opinion that non-combustible nicotine sources - that are significantly less harmful than conventional cigarettes - can be a viable solution for those who, for whatever reason, cannot or do not want to give up nicotine, or who want to cut back on smoking or quit altogether. Therefore, switching to combustion-free products has the potential to act as a gateway out of smoking. The personal preference for a particular product (e.g. e-cigarette vs HTP vs smokeless tobacco products) can play a critical role in increasing the likelihood of successfully abstaining from cigarette smoking. In any case, former smokers already using and smokers intending to use HTPs should receive correct information about their risk-benefit ratio. As for e-cigarettes, health professionals should consider all the options available to a smoking patient and opt for the ones that provide the greatest probability of quitting for good, including HTPs. For many smokers, one possible outcome may be switching to combustion-free products use for the long-term, while tolerating the small residual risk in return for a higher likelihood of success for smoking cessation and tobacco harm reduction.

## Abbreviations

CO: Carbon monoxide
CPCT: Centro per la prevenzione e cura del tabagismo
eCO: Exhaled breath carbon monoxide
ECs: Electronic cigarettes
GLO: Glo device with Kent Neostik, British American Tobacco
HTPs: Heated tobacco products
iQOS: iQos device with Marlboro Heets, Philip Morris International
NRT: Nicotine replacement therapies
PAHs: Polycyclic aromatic hydrocarbons
TSNAs: Tobacco-specific nitrosamines
